# Growth-inhibition patterns and transfer-factor profiles in arsenic-stressed rice (*Oryza sativa* L.)

**DOI:** 10.1007/s10661-017-6350-3

**Published:** 2017-11-16

**Authors:** Ha-il Jung, Jinwook Lee, Mi-Jin Chae, Myung-Suk Kong, Chang-Hoon Lee, Seong-Soo Kang, Yoo-Hak Kim

**Affiliations:** 1 Division of Soil and Fertilizer, National Institute of Agricultural Science, RDA, Wanju, 55365 Republic of Korea; 2 Department of Integrative Plant Science, College of Biotechnology and Natural Resource, Chung-Ang University, Anseong, 17546 Republic of Korea; 3 R&D Coordination Division, RDA, Jeonju, 54875 Republic of Korea

**Keywords:** Arsenic accumulation, Arsenic stress, Arsenic transfer factor, Growth inhibition, Potassium, Rice (*Oryza sativa* L.)

## Abstract

Arsenic (As) accumulation in rice owing to uptake from the soil is a critical human health issue. Here, we studied the chemical properties of As-treated soils, growth inhibition patterns of As-stressed rice plants, changes in the As content of soil and soil solutions, and the relationship between As accumulation and As transfer factor from the soil to the rice organs. Rice plants were cultivated in a greenhouse under four concentrations of As: 0 (control), 25, 50, and 75 mg kg^−1^. A significant positive correlation was found between available P_2_O_5_ and exchangeable K and between As concentration and available P_2_O_5_ or exchangeable K. The As concentration for 50% shoot growth inhibition was 50 mg kg^−1^. As levels in roots and shoots were positively correlated with the growth stages of rice. The transfer factor (TF)_root/soil_ increased with As concentration at the tillering stage but decreased at the heading stage. TF_root/soil_ and TF_shoot/soil_ were higher at the heading stage than at the tillering stage. As accumulation in the 25 mg kg^−1^ treatment was higher during the heading stage, whereas no difference was found at the tillering stage. As accumulation was related to plant biomass and soil As concentration. We found that As accumulation was greater at As concentrations that allowed for plant growth and development. Thus, species-specific threshold concentrations must be determined based on As phytotoxicity for the phytoremediation of As-contaminated soils. Hence, developing practical approaches for managing safe crop production in farmlands with an As contamination of 25 mg kg^−1^ or less is necessary.

**Electronic supplementary material:**

The online version of this article (10.1007/s10661-017-6350-3) contains supplementary material, which is available to authorized users.

## Introduction

Environmental toxicity owing to arsenic (As) contamination in arable lands causes major problems worldwide. Over the past few decades, millions of people in Southeast Asia have experienced As poisoning owing to consumption of contaminated rice (Bhattacharya et al. 2007; Mondal and Polya 2008; Phan et al. 2014). Environmental pollution, which is rapidly increasing owing to industrialization and urbanization, adversely affects human health. Anthropogenic activities, such as mining and smelting, have resulted in the contamination of agricultural irrigation water and the release of large amounts of As into paddy soils (Khan et al. 2010; Kim et al. 2016; Zhu et al. 2008). The primary determinant of As distribution is its naturally occurring concentration, but social and industrial activities play a primary role in the aggressive expansion of contaminated regions. Since the As contamination of rice has adverse long-term effects on health, rising levels of contamination are a matter of considerable importance (Chowdhury et al. 1999; Roychowdhury et al. 2002; Williams et al. 2005). In Korea, as well as in the rest of Southeast Asia, rice has been a dietary staple for millennia. This region not only accounts for 95% of the global rice production, but also has the highest level of rice consumption (Akinbile et al. 2011; Akinbile and Haque 2012; Kim et al. 2016). Thus, As contamination in rice is a critical issue for human health.

In comparison to other cereal crops (Sharma et al. 2016; Su et al. 2010; Williams et al. 2007), rice is cultivated in the reducing, anaerobic conditions of paddy soil, which results in more efficient absorption of As. Furthermore, when rice is grown in As-contaminated paddy soils, a high concentration of As accumulates in the plants and is transported to edible parts, such as the seeds, or to inedible organs, such as the root, stem, and leaf. This accumulation can result in serious health risks to the end consumer (Abedin et al. 2002; Jung et al. 2015; Xie and Huang 1998). Numerous studies have shown that the absorption, transport, and accumulation of As in rice plants differ depending on the rice cultivar and soil type (Ahmed et al. 2011; Chen et al. 2015; Liu et al. 2005; Norton et al. 2009; Ye et al. 2012). Moreover, using correlation studies, several researchers have clearly demonstrated an antagonistic interaction between As and phosphorus (P), which is one of the three major constituents of fertilizers (Cao et al. 2003; Gunes et al. 2009; Jiang et al. 2014; Meharg et al. 1994; Meharg and Macnair 1992). However, fewer studies have examined the interaction between As and potassium (K) (Carbonell et al. 1998; Tu and Ma 2005; Umar et al. 2013). Therefore, information on the interactions between As and K in rice is essential to further elucidating the effects of increased As on rice plants.

The absorption, translocation, and bioavailability of As in paddy soil have been reported to be closely related to pH, organic matter (OM), redox potential, and clay content. Therefore, these are important factors in predicting changes in the accumulation of As in rice (Bhattacharya et al. 2010; Meharg and Rahman 2003; Ye et al. 2012). However, there is a lack of standardization in the quantitative level of growth inhibition relative to the extent of As contamination. Similarly, the level of As accumulation and transport rate of As from the soil to the rice tissues at each stage of plant growth are uncertain. Research on the relationship between As concentration and the transfer factor and comparisons of changes in the soil based on soil As concentration are scarce. Quantifying the inhibition of growth as a result of different As concentrations and comparisons of the As transfer rate to different plant parts would enable predictions of As accumulation and would contribute to the production management and provision of safe agricultural products from As-contaminated farmlands.

Therefore, the present study was conducted with the following objectives: (1) to quantify the inhibition of plant growth relative to As concentration, (2) to examine the associations of soil As concentration with P and K, (3) to verify changes in As content in the soil solution relative to soil As concentrations, and (4) to evaluate the relationship between the As transfer factor and As accumulation from the soil to the different parts of the rice plant.

## Materials and methods

### Plant material and growth conditions

Rice (*Oryza sativa* L. “Dasan”) seeds were surface-sterilized in 0.2% Agri-Mycin solution (commercial bactericide, 8% ipconazole; Farm Hannong Co., Ltd. Seoul, Republic of Korea) for 48 h at ambient temperature and rinsed thoroughly with water. The surface-sterilized seeds were germinated under moist conditions (i.e., seeds were covered with two layers of moist paper towel) at 33 °C for 48 h. The germinated seeds were sown in trays (60 cm × 30 cm × 3 cm) filled with commercial potting soil (Pungnong Co., Ltd. Seoul, Republic of Korea) and grown in a greenhouse (National Institute of Agricultural Science, Wanju, Republic of Korea) during the rice-growing season (mid-June to late September) with natural sunlight and day/night temperatures of 30/25 °C with relative humidity of 60/80%, respectively. After 25 days, the seedlings were transplanted into Wagner pots (0.02-m^2^ surface area) filled with 3.5 kg of air-dried paddy soil and grown under flooded conditions (i.e., 3–4 cm of water above the soil surface).

### Experimental design

The pots were arranged in the greenhouse in a randomized complete block design with three replicates. Urea fertilizer was applied at 110 kg N ha^−1^, split into two applications: 50% pre-plant-incorporated (PPI) and 50% at the five-leaf stage. Phosphorus (45 kg ha^−1^) and potassium (57 kg ha^−1^) fertilizers were applied at PPI. The four levels of As applied were 0 (control), 25, 50, and 75 mg kg^−1^. According to Korean soil contamination criteria, As levels exceeding 25 and 75 mg kg^−1^ are the warning and action criteria, respectively. High As-contaminated soil (1050 mg As kg^−1^) was thoroughly mixed with the paddy soil to achieve the required As concentrations. The chemical properties of the soils are listed in Table 1.

**Table 1:** Chemical properties of the soil analyzed before the transplantation of rice seedlings and after harvesting rice plants at the heading stage

As (mg kg^−1^)	pH (1:5)	EC (dS m^−1^)	OM (g kg^−1^)	Av. P_2_O_5_ (mg kg^−1^)	Ex. cations (cmol kg^−1^)			
					K	Ca	Mg	Na
Before transplanting								
0	7.26a	0.37a	17.2a	77.3a	0.43a	6.87a	2.58a	0.14a
25	7.28a	0.39a	17.2a	76.4a	0.44a	6.97a	2.59a	0.16a
50	7.30a	0.38a	16.4a	80.0a	0.44a	7.11a	2.63a	0.17a
75	7.30a	0.37a	16.6a	78.0a	0.42a	7.20a	2.65a	0.19a
After harvesting								
0	7.76a	0.62a	16.4a	65.9d	0.28d	6.81a	2.94a	0.26a
25	7.62a	0.55a	16.3a	70.1c	0.38c	6.72a	2.65a	0.24a
50	7.60a	0.52a	16.6a	73.8b	0.41b	6.66a	2.55a	0.24a
75	7.54a	0.58a	16.1a	83.8a	0.46a	6.70a	2.56a	0.27a

Means within a column followed by the same letters are not significantly different at the 5% level according to Fisher’s least significant difference (LSD) tests

### Sampling of soil, soil solutions, and rice plants

Approximately 200 g of soil from each pot was collected before transplanting rice seedlings and after harvesting rice plants at the heading stage. The soil samples were air-dried, homogenized, sieved through a 2-mm mesh sieve, and stored at ambient temperature until further analyses of chemical properties. For collecting soil solution samples from the pots, soil moisture samplers (diameter 2.5 mm, membrane pore size 0.12–0.15 μm, and porous section 10 cm; Rhizosphere Research Products, Wageningen, The Netherlands) were vertically inserted to a 10-cm depth in the pots. Soil solution samples were collected using a syringe before transplanting rice seedlings and at the rice heading stage. The samples were stored in 50-mL polypropylene tubes at 4 °C until further As analyses. For each treatment, three pots were destructively harvested at the rice tillering and heading stages. The harvested root and shoot samples were first thoroughly washed with tap water and then with deionized water to remove dust and adhering soil particles. The washed samples were then dried at 60 °C for 72 h in an oven and subsequently powdered. The powder was stored in 50-mL polypropylene tubes at ambient temperature until As analysis.

### As content of soil, soil solutions, and rice tissues

All samples used in this study were analyzed based on the established protocols for soil and plant analyses (NAAS (National Academy of Agricultural Science) 2011). Soil pH and electrical conductivity (EC) were measured using a 1:5 mixture of soil and distilled water. OM content was determined using a wet digestion method, available phosphate (Av. P_2_O_5_) was calibrated using the Lancaster method (Cox 2001), and exchangeable cations were determined using 1 M ammonium acetate (pH 7).

The chemical properties of the soil analyzed before transplantation of rice seedlings and after harvesting of rice plants at the heading stage are listed in Table 1. The As content of the soil was analyzed using an inductively coupled plasma-optical emission spectrometer (ICP-OES; GBC AU/Integra XL, GBC Scientific Equipment Ltd., Melbourne, Australia). The homogenized soil (3 g) was digested using a Gerhardt trace metal digestion system (SMA-20A, Gerhardt Co., Ltd. Königswinter, Germany), cooled to ambient temperature, diluted to 100 mL with ultrapure water, and filtered through Whatman No. 40 filter paper (Whatman, Buckinghamshire, UK) into a 100-mL volumetric flask. This filtrate was used for ICP-OES spectrometry.

Inductively coupled plasma-mass spectroscopy (ICP-MS; Agilent 7900, Agilent Technologies Inc., Santa Clara, USA) was used to analyze As content in soil solutions. The soil solution (10 mL) was digested using a graphite block acid digestion system (ODLAB Co., Ltd. Seoul, Republic of Korea). After digestion, the solution was cooled to ambient temperature and diluted to 50 mL with ultrapure water. This diluent was used for the ICP-MS spectrometry.

For measuring As content in the roots and shoots of rice plants, 200 mg of powdered sample was digested using the graphite block acid digestion system. After digestion, the solutions were cooled to ambient temperature, diluted to 100 mL with ultrapure water, and filtered through Whatman No. 40 filter papers. This filtrate was used for ICP-MS spectrometry.

### Plant growth response and analysis

The effects of As on plant height, tiller number, shoot dry weight (DW), and shoot water content (WC) of rice plants were measured at the tillering and heading stages (Table 2). The plants were cut at the soil surface, and shoot fresh weights were recorded immediately for calculating shoot water content. The shoots were then dried at 60 °C for 72 h and weighed again to obtain the shoot dry weight.

**Table 2:** Effect of As treatments on plant height, tiller number, shoot dry weight, and shoot water content of rice plants at the tillering and heading stages

As (mg kg^−1^)	Plant height (cm)	Number of tillers	Shoot dry weight (g)	Shoot water content (%)
Tillering stage				
0	72a	17.0a	15.3a	76a
25	70a	15.0ab	12.0ab	78a
50	62b	14.0b	10.4b	77a
75	64b	11.3c	7.5c	76a
Heading stage				
0	97a	17.0a	52.4a	67b
25	90b	12.0b	40.8b	66b
50	70c	12.7b	23.2c	71a
75	60d	10.7b	14.9d	70a

Means within a column followed by the same letters are not significantly different at the 5% level according to Fisher’s least significant difference (LSD) tests

### Data analysis

**Fig. 1:**
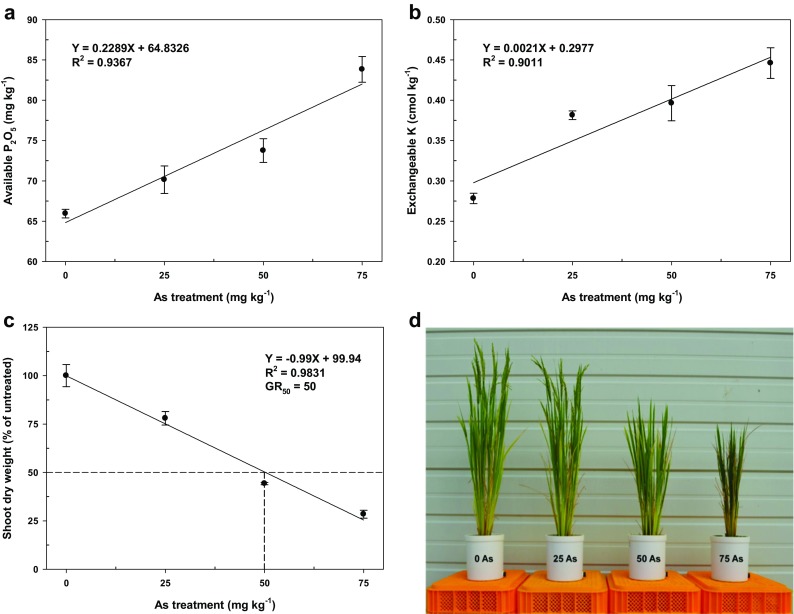
Relationship between As concentrations and available P_2_O_5_ (**a**) and exchangeable K (**b**) contents at the heading stage of rice and the effect of As treatments on shoot dry weight (**c**) and growth inhibition (**d**) of rice plants at the heading stage. The GR_50_ value is the As concentration that reduced shoot dry weight by 50%. Vertical bars represent standard deviations of the mean

The relationships between As concentrations and Av. P_2_O_5_ (Fig. 1a) and exchangeable (Ex.) K (Fig. 1b) and between Av. P_2_O_5_ and Ex. K (Fig. S1) were evaluated by linear regression analyses after harvesting rice plants at the heading stage.

For the evaluation of growth inhibition, data were expressed as the percentage of the mean growth of untreated rice plants in order to standardize comparisons between the As treatments. A linear regression was used to describe growth inhibition at the heading stage. The quantity of As that would reduce shoot dry weight by 50% (GR_50_) was calculated from the regression equation (Fig. 1c).

To evaluate the relationship between the As contents of the roots and shoot As content of rice plants at tillering (Fig. 3c) and heading (Fig. 3d) stages, a linear regression was performed on the data obtained from the four As treatments.

The soil to root (Fig. 4a) or shoot (Fig. 4b) transfer factor (TF) was calculated as follows:

$$ \mathrm{TF}=\frac{Ca}{Cb} $$

where *Ca* is the As concentration of the root or shoot and *Cb* is the As concentration of the soil.

To elucidate the physiological responses of rice plants to varying levels of As, a principal component analysis (PCA) and a correlation coefficient analysis were performed using MetaboAnalyst 3.0 (www.metaboanalyst.ca) for cluster analysis and heat map generation. Ward’s clustering algorithm and Pearson’s distance were applied for cluster analysis.

All data obtained were subjected to an analysis of variance (ANOVA). To identify significant differences between treatments, Fisher’s least significant difference (LSD) tests were performed with a statistical analysis software (SAS ver. 9.2, SAS Institute Inc., Cary, NC, USA). Differences at *P* < 0.05 were considered significant. Data in figures are expressed as mean values ± standard deviations (SD).

## Results and discussion

### Changes in the chemical properties of As-treated soils

The chemical properties of the potting soil treated with 0, 25, 50, or 75 mg As kg^−1^ were analyzed from the samples collected immediately before transplanting (pre-experiment) and at the heading stage (post-experiment) (Table 1). No significant differences were noted in the chemical properties between the pre- and post-experimental periods, with the exception of Av. P_2_O_5_ and Ex. K. Although no significant differences between treatments were observed before the experiment, the concentrations of Av. P_2_O_5_ and Ex. K were significantly different after the experiment (Table 1).

As the concentration of As increased, Av. P_2_O_5_ (Fig. 1a) and Ex. K (Fig. 1b) increased proportionally in soil, where significant positive correlations between As concentration and Av. P_2_O_5_ (Fig. 1a) and Ex. K (Fig. 1b), as well as between Av. P_2_O_5_ and Ex. K (Online Resource 1), were observed. To the best of our knowledge, this is the first result to demonstrate the association of As with K in soil. Although the result of the plant analysis showed the association of As with K and P, Liu et al. (2008) reported significant decreases in total K and P concentrations in As treatments of both the shoots and roots of *Triticum aestivum* under hydroponic conditions. P can affect As uptake by rice, which can be explained by the effect of P on the adsorption of As onto soil particles and the subsequent solubility and bioavailability of As for uptake by rice (Gupta and Ahmad 2014; Islam et al. 2016; Panda et al. 2010). Meharg and Macnair (1992) reported a negative relationship between P and arsenate uptakes by *Holcus lanatus* roots. Our results were similar to those of Umar et al. (2013), who reported low As uptake by garden cress when low concentrations of K were applied (20–40 mg L^−1^) but high As uptake when higher concentrations of K were applied (60–100 mg L^−1^) under hydroponic growth conditions. In addition, Carbonell et al. (1998) and Tu and Ma (2005) showed that K competes with As for uptake by plants. These results show that P and K fertilizers can act as important factors in the growth and development of crops cultivated in fields contaminated with relatively low As concentrations (25 mg kg^−1^ and lower) and suggest that the management of P and K fertilizations can be used to control As absorption and reduce plant toxicity.

### Growth inhibition patterns in As-stressed rice

As toxicity adversely affects photosynthesis by reducing chlorophyll content, thereby negatively affecting overall growth, vital metabolic processes, and crop yields (Gupta and Ahmad 2014; Panda et al. 2010). In the present study, we found that rice plants exhibited an As concentration-dependent inhibition of growth and development. Excluding shoot water content, the presence of As resulted in a reduction in plant height, number of tillers, and shoot dry weight at both the tillering and heading stages in rice plants. Although shoot water content did not differ between the tested As levels at the tillering stage, shoot water content was higher at 50 and 75 mg As kg^−1^ than at 0 and 25 mg As kg^−1^ at the heading stage (Table 2). To quantify growth inhibition in relation to As levels, shoot dry weight, a parameter that shows growth inhibition proportional to As concentration, was used in a regression analysis (Fig. 1c). Plant growth decreased linearly with increased As concentrations, and emergence and heading were essentially inhibited at As levels higher than 50 mg kg^−1^ (Fig. 1d). Thus, an As concentration of 50 mg kg^−1^ caused a 50% inhibition in growth owing to plant toxicity. The regression analysis showed that a 1% inhibition in rice growth was inversely proportional to a 1 mg As kg^−1^ increase in As concentration (Fig. 1c).

### Changes in the As content of soil and soil solutions

**Fig. 2:**
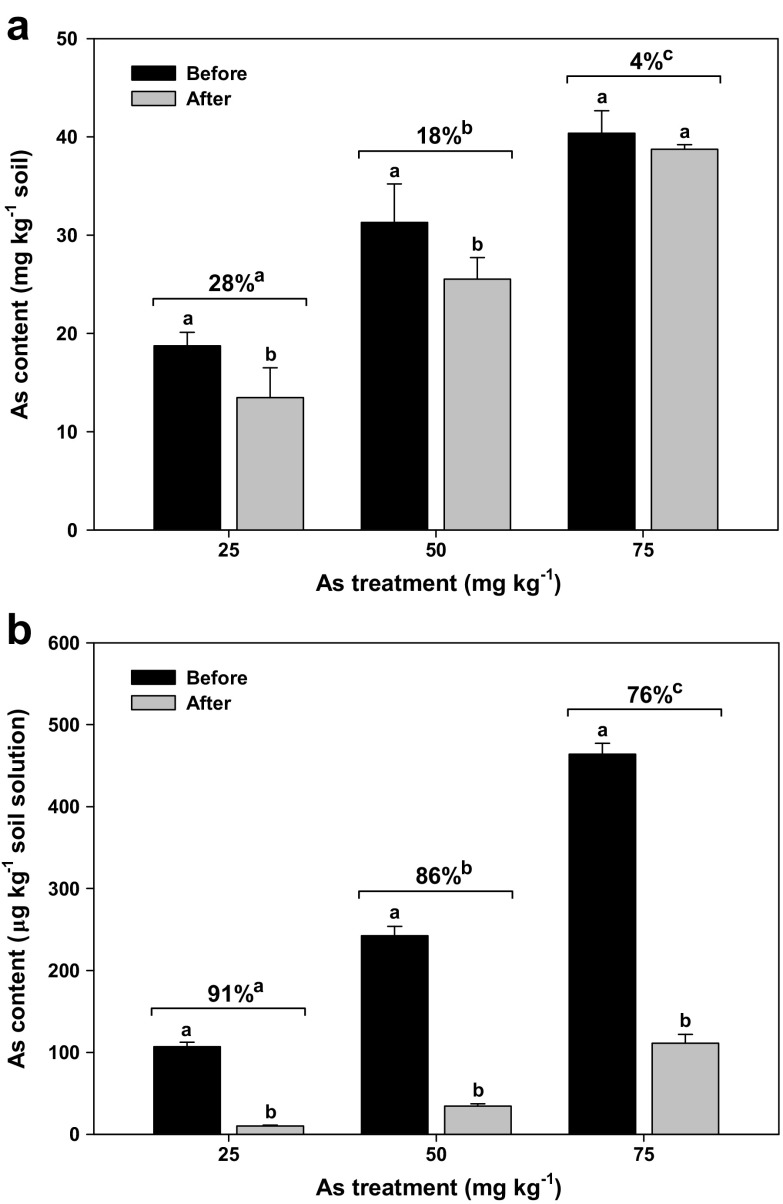
Changes in As content of the soil (**a**) and soil solutions (**b**) before transplanting rice seedlings (before) and after harvesting rice plants at the heading stage (after) and the reduction rates (numbers at the top of the columns) of As content. Vertical bars represent standard deviations of the mean

The As content of the soil samples treated with 25, 50, and 75 mg As kg^−1^ decreased by 28, 18, and 4%, respectively (Fig. 2a), and those of the soil solutions were reduced by 91, 86, and 76%, respectively (Fig. 2b). In addition, the rate of decrease in As content was the highest at 25 mg As kg^−1^, followed by 50 and 75 mg As kg^−1^ (Fig. 2a, b). This could be because the maximum limit of As absorption varies with plant species, cultivars, growth stages, and environmental conditions. When the As concentration is higher than the maximum absorption limit, As absorption decreases owing to overall growth inhibition in plants (Panda et al. 2010; Warren et al. 2003).

### Relationship between As contents in different organs

**Fig. 3:**
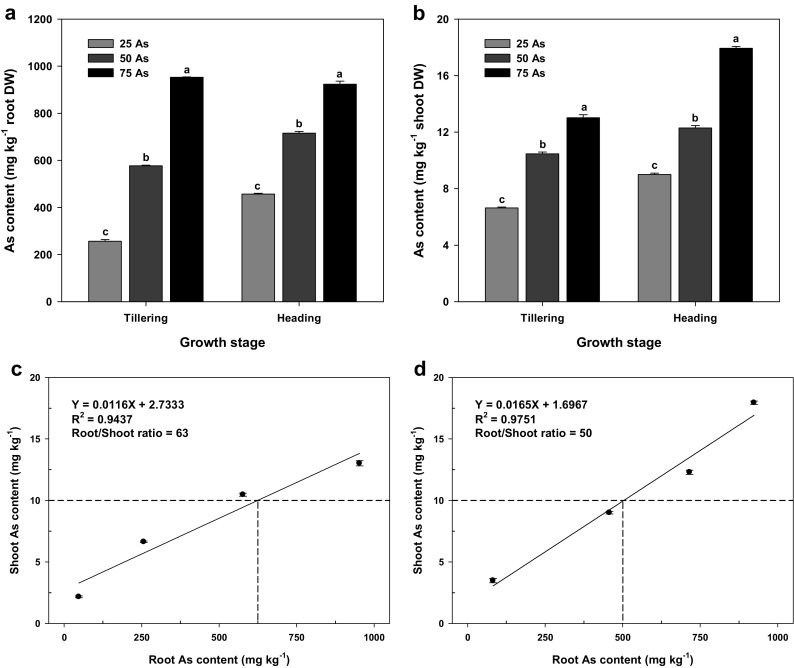
Effect of As treatments on As content in the roots (**a**) and shoots (**b**) and the relationship between root and shoot As contents of rice plants at the tillering (**c**) and heading (**d**) stages. Vertical bars represent standard deviations of the mean

The As content in roots and shoots increased proportionally with As concentration at both the tillering and heading stages, where As levels were higher in the roots than in shoots (Fig. 3a, b). The results showed a positive correlation between As content in roots or shoots and the growth stages of rice. The root/shoot ratio at the tillering stage was 63 (Fig. 3c), which was higher than the ratio of 50 observed at the heading stage (Fig. 3d). Thus, a relatively greater amount of As was absorbed and sequestered in the roots at the tillering stage than at the heading stage. Subsequently, as plant biomass increased, an increase in the transfer of As to the shoot occurred.

### As transfer-factor profile and accumulation in As-stressed rice

**Fig. 4:**
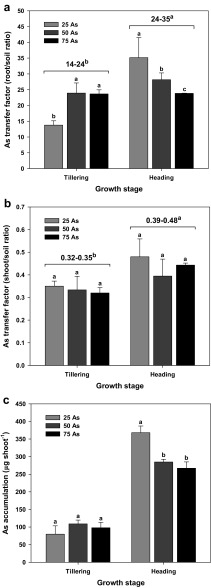
Effect of As treatments on As transfer factors between the soil and the root (**a**) or shoot (**b**) and As accumulation (**c**) in rice plants at the tillering and heading stages and a comparison of the transfer factors between tillering and heading stages (letters at the top of the columns). Vertical bars represent standard deviations of the mean

In contrast to the increase in TF_root/soil_ observed with increasing As treatment at the tillering stage, a decreasing trend was observed at the heading stage (Fig. 4a) (Gupta and Ahmad 2014). The TF_root/soil_ at the heading stage was significantly higher than that at the tillering stage (Fig. 4a). However, no significant differences were observed for TF_shoot/soil_ between As treatments at the different growth stages of rice (Fig. 4b). TF_shoot/soil_ was higher at the heading stage than at the tillering stage (Fig. 4b). These results are similar to those of Williams et al. (2007), who reported 50-fold higher values for wheat and barley. The TF_root/soil_ increased as the As content of the soil increased. In contrast, TF_shoot/root_ decreased with increasing As levels in the soil. Similarly, all other TFs (grain/soil, grain/root, and shoot/soil) either remained unchanged or decreased with increasing soil As levels (Batista et al. 2014). Overall, these results clearly show (1) that the shoot transfer rate of As was maintained at a constant value, regardless of the As level, and (2) that the growth stage of the plant and not the As concentration of the soil affected the TF values (Batista et al. 2014; da Silva et al. 2016; Gupta and Ahmad 2014).

Since the estimation of As accumulation in the shoot at different growth stages was based on biomass, As accumulation was significantly higher at the heading stage, when the plants have undergone considerable growth, in comparison to plants in the tillering stage, which experienced a relatively short As exposure period. Furthermore, As accumulation in rice plants was found to be the highest at the lowest As treatment concentration of 25 mg As kg^−1^, where no significant differences were observed between the higher treatments at 50 and 75 mg As kg^−1^ (Fig. 4c). These results indicate that As accumulation was highly dependent upon plant biomass and not dependent on As concentration in the soil. Thus, it can be concluded that As accumulation by the rice plants was more efficient when the As concentration in the soil allowed better plant growth and development.

### Principal component analysis of the physiological responses of rice plants

**Fig. 5:**
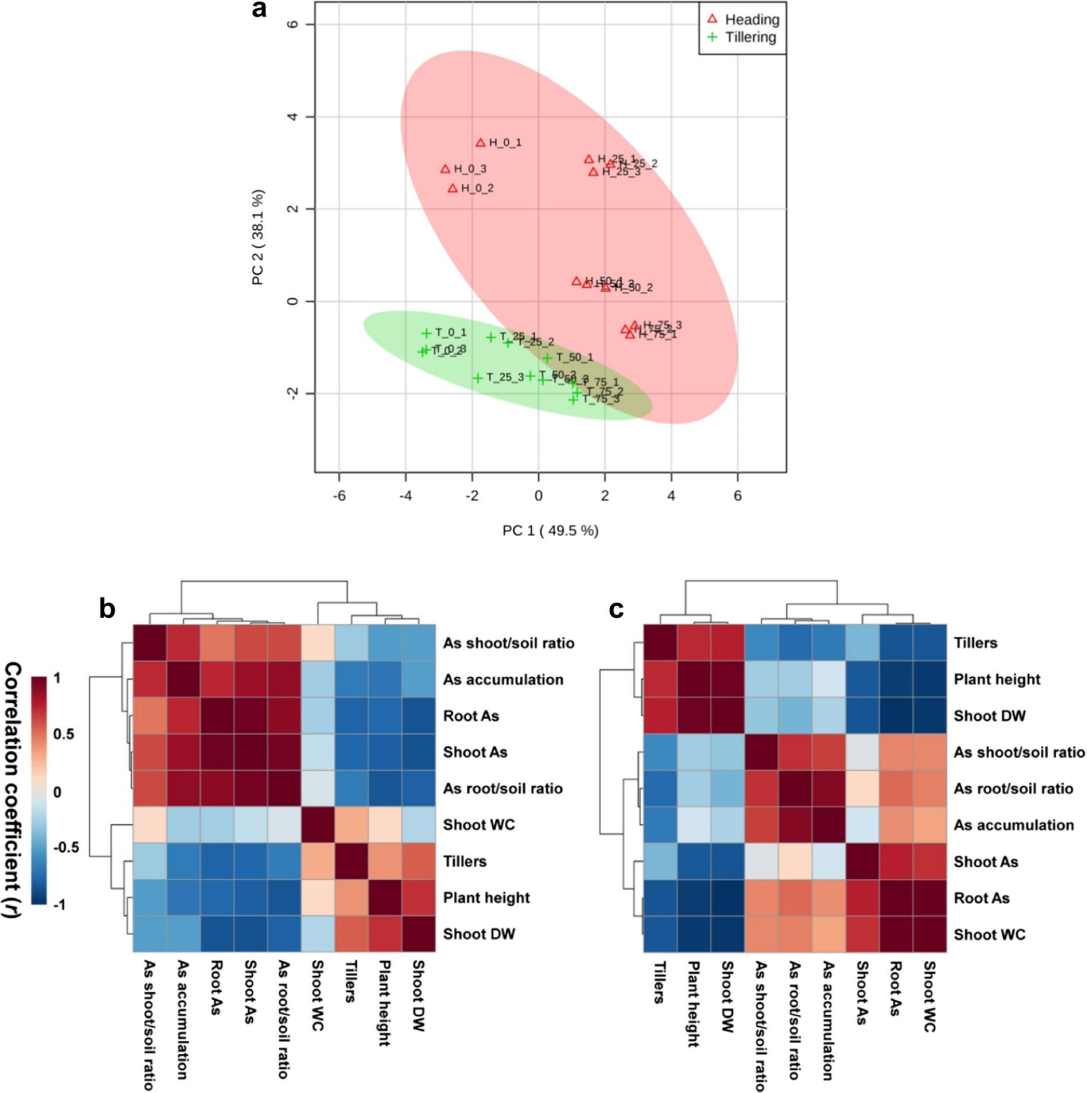
Principal component analysis (PCA) score plot (**a**) and heat map responses of Pearson’s correlation coefficient (*r*) for the physiological responses of the growth characteristics of rice plants to applied As levels at the tillering (**b**) and heading (**c**) stages. *T* tillering stage, *H* heading stage. The numbers after T or H indicate As concentration (0, 25, 50, or 75 mg As kg^−1^), while the final digit indicates which of the three replicates is shown

A principal component analysis (PCA) score plot was used to evaluate overall responses of rice plants to various levels of As at the tillering and heading stages (Fig. 5a). The PCA score plot accounted for 49.5 and 38.1% of the variance of principal component 1 (PC-1) and principal component 2 (PC-2), respectively. These results indicated that plant physiological responses to treatment with different As levels varied at both growth stages. A comparison of the physiological responses at these two growth stages showed that they were considerably different at the lower levels (0 and 25 mg kg^−1^) of applied As than at the higher levels (50 and 75 mg kg^−1^). Furthermore, this result showed that the heading stage was more responsive to As application than the tillering stage, as the response variables in the PCA score plot were much wider and more extensive at the heading stage than at the tillering stage.

At the tillering stage, As shoot/soil ratio, As accumulation, root As, shoot As, and As root/soil ratio were positively correlated with each other but were negatively correlated with tiller number, plant height, and shoot DW (Fig. 5b). At the heading stage, three large positive correlation clusters were identified: (1) tillers, plant height, and shoot DW; (2) As shoot/soil ratio, As root/soil ratio, and As accumulation; and (3) shoot WC, root As, and shoot As. Furthermore, tillers, plant height, and shoot DW were negatively correlated with the other variables (Fig. 5c). Therefore, these results indicate that plant growth stages would be highly affected by the application of As.

## Conclusion

Rice growth decreased linearly with As concentration, where the reduced growth inhibited the absorption of the macro-elements P and K, both of which are essential to plant growth and development. Our results clearly show that As transfer to the shoot increased with increases in the duration of As exposure and increased with plant growth and development but was not affected by the As content of the soil. Moreover, As accumulation in plants decreased when As concentrations exceeded a threshold concentration for plant growth. Therefore, for the phytoremediation of As-contaminated soils, the threshold limit concentration for plant growth and development should be determined based on As toxicity in different plant species. Furthermore, our findings highlight the need to develop management methods for safe crop production in farmlands with relatively low As contaminations levels of 25 mg kg^−1^ or less.

## Electronic supplementary material

ESM 1 (DOCX 53 kb).
