# Supplementary material for: Growth-inhibition patterns and transfer-factor profiles in arsenic-stressed rice (Oryza sativa L.)
Source: Environ Monit Assess. 2017 Nov 16;189(12):638. doi: 10.1007/s10661-017-6350-3 (PMC5691118; doi:10.1007/s10661-017-6350-3)
Supplement: Supplementary file 1 — (DOCX 53 kb). [file 10661_2017_6350_MOESM1_ESM.docx]

**Fig. S1** Relationship between available P_2_O_5_ (A) and exchangeable K contents at the heading stage of rice. Vertical bars represent standard deviations of the mean.
